# Grading of Balance Function in Subacute Stroke Patients by Using the Berg Balance Scale Together with Latent Rank Theory

**DOI:** 10.1298/ptr.E10282

**Published:** 2024-05-10

**Authors:** Shuntaro TAMURA, Kazuhiro MIYATA, Sota KOBAYASHI, Ren TAKEDA, Hiroki IWAMOTO

**Affiliations:** ^1^Department of Rehabilitation, Fujioka General Hospital, Japan; ^2^Department of Physical Therapy, Ibaraki Prefectural University of Health Sciences, Japan; ^3^Department of Rehabilitation, Public Nanokaichi Hospital, Japan; ^4^Department of Basic Rehabilitation, Gunma University Graduate School of Health Sciences, Japan; ^5^Department of Rehabilitation, Day Care Center Specialized in Stroke Rehabilitation “With Reha”, Japan; ^6^Department of Rehabilitation, Hidaka Rehabilitation Hospital, Japan

**Keywords:** Latent rank theory, Berg Balance Scale, Balance, Early subacute stroke, Outcome measure

## Abstract

Objectives: The Berg Balance Scale (BBS) is a core measure of balance function in patients with stroke. Latent rank theory (LRT) is a statistical method that enables the degree of functional impairment to be ranked from the sub-items of a rating scale; each rank can then be characterized. Identification of the characteristics of balance function by rank would be beneficial for interventions to improve balance function in patients with stroke. This study aims to use LRT to rank and characterize patients with stroke balance impairment. Methods: This was a multicenter retrospective analysis of 293 patients with subacute stroke. We used LRT and the BBS to estimate the optimal rankings based on the goodness-of-fit index and the information criterion. We compared the obtained ranks with the level of walking independence for each rank. Results: The evaluation of the patient’s BBS scores revealed that balance impairment could be divided into six ranks. The average BBS score for each rank rose from 27.1 for rank 1 to 53.9 for rank 6. The scores of the BBS sub-items for each rank also differed. The level of walking independence by rank ranged from rank 1 for assisted walking to rank 6 for independent outdoor walking. Conclusions: Balance function in patients with subacute stroke was ranked sixth in the BBS, with varying characteristics identified for different ranks. This result helped to determine the therapy to improve the balance function of patients with stroke.

## Introduction

Balance impairment is common among individuals who have experienced a stroke, and this impairment can have significant impacts on functional independence. The control of balance is complex, requiring the maintenance ofposture, the promotion of movement, and the restorationof equilibrium, and there are many different types of balance problems^1)^. Walking independence has a significant influence on a person’s ability to engage in his or her activities of daily living independently, and sufficient balance function is important for walking independence^2^^,^^3)^. It is therefore important to (i) evaluate the balance function of patients who have experienced a stroke and (ii) assure that the patient’s balance function is reflected in goal setting and treatment content.

The Berg Balance Scale (BBS) is a core outcome measure for measuring adults’ balance in both research and clinical practice^4)^. The BBS also assesses balance function, one of the core outcome measures for individuals with neurological disorders^5)^. It is a simple and easy assessment that does not require the use of special tools, and it is frequently used in the evaluation of patients with stroke^6)^. The BBS has been reported to have high internal consistency (Cronbach’s α = 0.92–0.98) and reliability (intra-class correlation coefficient = 0.95 and 95% confidence interval = 0.93–0.97) in patients from early stroke onset up to 180 days^7)^. An excellent correlation (r = 0.76) has also been reported between the BBS and the functional independence measure in patients with stroke who are in inpatient rehabilitation^8)^.

Several cutoff values of the BBS have been indicated for walking independence^9–11)^. Since the cutoff values are used as reference values to classify patients into two groups, they do not provide information on the characteristics of a given patient’s balance impairment. Therefore, the BBS cutoff values alone do not provide sufficient information to treat a patient. In addition, the sub-items of the BBS have been found to vary in difficulty^12–14)^. Although these studies indicate differences in achieving a perfect score on the BBS sub-items, they do not reveal differences in the difficulty levels of individual scores within these sub-items. It is thus important to determine the degree of balance impairment based on the total BBS score and to identify the characteristics of balance impairment based on the sub-item scores. The characteristics of the BBS sub-items for each degree of balance impairment can be compared with the subject’s BBS sub-items to identify balance practice issues that should be prioritized.

Latent rank theory (LRT) can be used to classify the severity of impairment using information about the distribution of scores and the total scores of the sub-items of a rating scale and to identify the functional impairment characteristic of each rank of severity^15^^,^^16)^. In the field of education, for example, LRT is used in multi-question surveys to clarify the characteristics of the target population^17)^. Although LRT was developed in the field of education, its application in rehabilitation can be used to clarify the structure of various functional disabilities to be treated. The use of LRT for the BBS, a comprehensive balance function evaluation, enables us to rank the severity of balance impairment and to identify the characteristics of each rank. In addition, it is possible to identify sub-items of the BBS that require treatment to achieve a higher rank of balance function and to help develop a treatment plan. In other words, items showing a discrepancy between the characteristics of the balance function’s sub-items, as estimated rank from the subject’s total BBS score, and the actual scores of the subject’s sub-items can be targeted for therapy. This means that when physical therapy is conducted to improve a patient’s balance, intervention plans can be considered in greater detail according to the severity of the impairment.

In patients with stroke, the first 6 months after onset are the most likely time for functional improvement^18^^,^^19)^. Characterizing the balance impairment in patients with stroke during this phase may thus be useful for determining the optimal therapy. We conducted this study using the BBS to classify and characterize the severity of balance impairment in patients with subacute stroke.

## Methods

This retrospective multicenter observational study included inpatients with stroke admitted to four hospitals in Japan that provide rehabilitation for patients with stroke. The period for data collection was between April 2018 and June 2020. This study was approved by the Ethical Review Committees of Fujioka General Hospital, Nanokaichi Public Hospital, Hidaka Rehabilitation Hospital, and Numata Neurosurgery & Heart Disease Hospital (approval nos. #194, #20200020, #20200503, and #00032). It was conducted in accordance with the Declaration of Helsinki. Only the patients’ existing data were used for the analyses, and information about the study, including opt-out options, was made publicly available. The patients were provided an opportunity to withdraw from participation in the study.

### Patients

Data were collected from the patient’s medical records. The inclusion criteria were individuals aged ≥20 years who were admitted to one of the four hospitals with a first supratentorial stroke and discharged within 6 months of the onset of the stroke. The exclusion criteria were as follows: (1) inability to complete an accurate assessment of balance and gait function with verbal instructions, (2) diagnosis of a neurological disease other than stroke, (3) a missing BBS score on discharge, and (4) declining to be included in the study.

The patients’ physical function was assessed before discharge. Their balance function was assessed by the BBS, and their gait function was assessed by the functional ambulation categories (FACs) test. Each patient’s use of any walking aid was also recorded. Physical therapy during admission included muscle-strengthening training, balance training, and level gait training. If the patient was able to walk safely on level ground, more difficult exercises such as stair climbing and walking on rough ground were carried out.

### Assessment tools

The BBS is a 14-item comprehensive measure of static and dynamic balance function. Sub-items are scored on a five-point ordinal scale from 0 to 4, with a maximum score of 56^20)^. The BBS has shown no significant floor or ceiling effect before or after inpatient rehabilitation in patients with subacute stroke^21)^. The FAC test classifies the level of walking independence with and without physical aids on a six-point scale from 0 to 5, ranging from inability to walk (0 points) to walking independently outdoors (5 points)^22)^. The FAC test is a brief method of measuring a person’s gait; it uses a straight walking path and stairs, and it has shown high test–retest reliability (Cohen’s κ = 0.95) and very good inter-rater reliability (κ = 0.91) in patients with subacute stroke. The FAC has also been correlated and validated with walking speed, stride length, and the six-minute walk test^23)^.

In the present patients, the motor function of the lower extremity on the paralyzed side was assessed at the Brunnstrom recovery stage (BRS)^24)^. The BRS of the lower extremity is classified from I (flaccid) to VI (isolated joint motion).

### Data analysis

The LRT is a statistical model that assumes the ordinality of variables; it evaluates a subject’s status in rank^25)^. Using the LRT, an item reference profile, a test reference profile, and a rank membership profile were obtained. The item reference profile is the expected score for each sub-item in the targeted assessment of the subjects belonging to each rank; it shows the rank-specific characteristics of each item. In LRT, the item level of difficulty for each sub-item is determined by the rank at which the item reference profile can obtain 50% of the score of the sub-item (here, 2.0 points for each sub-item of the BBS). An item is, therefore, considered to be more difficult if the item reference profile achieves 50% of the sub-item scores at a higher rank.

The test reference profile is the average expected score of a subject who belongs to each rank in the target evaluation; it is a weighted sum of the item reference profiles, which presents the average score of each rank. In the LRT, the evaluation scale must be ordinal, and the prerequisite for using the LRT is that the ranks must satisfy the weakly ordinal placement condition in which the test reference profiles increase monotonically with each rank as the condition for the ranks to be ordinal. The rank membership profile is the probability that the subject belongs to each rank. Even if a new evaluation is performed on a different subject, it is possible to estimate the rank to which the subject belongs by adding information to the dataset.

Two methods are used for an analysis with LRT: using the mechanism of a self-organizing map (SOM) and generative topographic mapping. Using the SOM is recommended when the number of data is <3000^25)^. We used an SOM in this study. For the analysis, the scores for each sub-item of the BBS were inputted. Although LRT can be used to analyze any number of ranks, the minimal detectable change (MDC) 90 of the BBS in patients with stroke is 6 points, and the MDC 95 is 7 points^26)^. The classification of >10 ranks is affected by measurement error. We considered the effect of measurement error to be large, and we thus specified the number of ranks for the BBS as 3 to 9.

The LRT enables us to specify the distribution of subjects per rank. The distribution can be specified as a not-specified distribution, a uniform distribution, and a normal distribution. To determine the model, we divided the prior distribution into 3 to 9 ranks with a not-specified distribution, a uniform distribution, and a normal distribution, and we calculated and compared the goodness-of-fit index and the information criterion. The goodness-of-fit index is expressed by the root mean square error of approximation (RMSEA), and the goodness-of-fit is considered to be <0.06^27)^. The information criterion is used to evaluate the relative performance of the model, and with it, Akaike’s information criterion (AIC), the consistent AIC (CAIC), and the Bayesian information criterion (BIC) are obtained. These are compared relative to each other on each index, and a model is considered to be good if each information criterion’s value is low. The choice of model was made based on the goodness-of-fit indices of the candidate models, with the candidate model being the one with the smallest value of AIC, CAIC, or BIC in each type of distribution.

To evaluate the external validity of the calculated balance function characteristics for each rank, we determined the distribution of the number of FACs in each rank. Spearman’s correlation coefficient between the ranks and FACs was also determined. The correlation coefficients were interpreted as follows: 0.00–0.25, little to no relationship; 0.26–0.49, fair correlation; 0.50–0.69, moderate correlation; 0.70–0.89, strong correlation; and 0.90–1.00, very strong correlation^28)^. All statistical analyses were performed using the Exametrika5.4 software package^25)^ and the R program ( ver. 4.1.0; R Foundation for Statistical Computing, Vienna, Austria). We used p <0.05 as the level of significance.

## Results

This retrospective study included 293 individuals with stroke. The characteristics of the patients are summarized in Table 1. The mean (SD) age of the subjects was 72.4 (12.3) years, the mean (SD) days since onset was 51.7 (48.2), and motor paralysis was mild. The degrees of walking independence were assisted walking (FAC ≤2) in 37 patients, supervised walking (FAC = 3) in 81 patients, and independent walking (FAC ≥ 4) in 175 patients.

**Table 1.** Characteristics of study participants (n = 293)

	Value
Age: years (mean ± SD)	72.4 ± 12.3
Gender (male/female)	195/98
Stroke type (ischemic/hemorrhagic)	240/53
Hemiplegic side (left/right)	148/145
BRS of lower extremity (II/III/IV/V/VI)	5/8/18/109/153
Time since stroke (days)
Mean ± SD	51.7 ± 48.2
Median (1st–3rd quartiles)	34.0 (12.0–81.0)
Walking aids (without/cane/walker)	246/32/15
FACs (0/1/2/3/4/5)	17/5/15/81/107/68
BBS score
Mean ± SD	45.8 ± 13.0
Median (1st–3rd quartiles)	50.0 (44.0–55.0)

SD, standard deviation; BRS, Brunnstrom recovery stage; FACs, functional ambulation categories; BBS, Berg Balance Scale

The goodness-of-fit indices and information criteria for each number of ranks for each distribution model are shown in Table 2. Of the models with the lowest AIC, CAIC, or BIC values, the lowest RMSEA was in the six-ranked models with a not-specified distribution. We thus used a six-rank model with a not-specified distribution in the further analyses.

**Table 2.** Goodness-of-fit index and information criteria for each number of ranks foreach distribution

Distributions	RMSEA	AIC	CAIC	BIC
Not specified				
3 Rank	0.044	−528.710	−**6191.718**	−**4981.718**
4 Rank	0.038	−672.945	−6078.545	−4923.545
5 Rank	0.033	−743.450	−5891.639	−4791.639
6 Rank	**0.031**	−**751.670**	−5642.450	−4597.450
7 Rank	**0.031**	−717.939	−5351.310	−4361.310
8 Rank	**0.031**	−677.158	−5053.120	−4118.120
9 Rank	0.032	−612.314	−4730.866	−3850.866
Uniformity distribution				
3 Rank	0.050	−319.668	−5982.677	−4772.677
4 Rank	0.046	−442.719	−5848.318	−4693.318
5 Rank	0.042	−529.652	−5677.842	−4577.842
6 Rank	0.046	−386.858	−5277.638	−4232.638
7 Rank	0.039	−542.158	−5175.529	−4185.529
8 Rank	0.041	−475.692	−4851.654	−3916.654
9 Rank	0.044	−389.522	−4508.074	−3628.074
Normal distribution				
3 Rank	0.058	−5.081	−5668.090	−4458.090
4 Rank	0.058	−31.165	−5436.764	−4281.764
5 Rank	0.047	−388.695	−5536.885	−4436.885
6 Rank	0.050	−288.656	−5179.436	−4134.436
7 Rank	0.046	−385.793	−5019.164	−4029.164
8 Rank	0.045	−384.841	−4760.803	−3825.803
9 Rank	0.048	−291.536	−4410.088	−3530.088

The lowest value for each indicator is shown in bold.

RMSEA, root mean square error of approximation; AIC, Akaike’s information criterion; CAIC, consistent Akaike’s information criterion; BIC, Bayesian information criterion

**Fig. 1.**
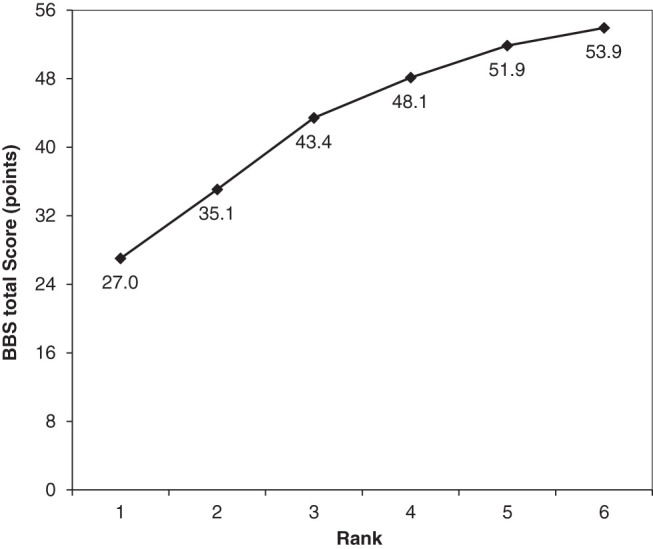
Test reference profile for six ranks with no distribution specified. The test reference profile is the average expected score of a subject who belongs to each rank. The horizontal axis indicates each rank from 1 to 6 in the six-rank model of not-specified distribution. BBS, Berg Balance Scale

As illustrated in Figure 1, the test reference profile increased with the six ranks in the following order: 8.1, 8.3, 4.7, 3.8, and 2.0 points. An essential condition for LRT was thus met; that is, the weak-order placement condition, in which test reference profiles monotonically increase with each rank. The number of patients in each rank was 41, 11, 44, 54, 34, and 109, in order from rank 1 to rank 6. The item reference profile of the BBS sub-items also increased with each rank (Table 3). In terms of the difficulty of the BBS items, sitting unsupported was the easiest, and standing on one leg was the most difficult.

**Table 3.** Item reference profile for six ranks with no distribution specified

BBS sub-item	Item reference profile (points)					
	Rank 1	Rank 2	Rank 3	Rank 4	Rank 5	Rank 6
Sitting unsupported	3.6	3.8	3.9	4.0	4.0	4.0
Sitting to standing	2.7	3.3	3.8	4.0	4.0	4.0
Standing to sitting	2.6	3.2	3.8	3.9	4.0	4.0
Standing unsupported	2.5	3.2	3.8	4.0	4.0	4.0
Standing with eyes closed	2.5	3.1	3.7	3.9	4.0	4.0
Transfers	2.4	3.0	3.6	3.9	4.0	4.0
Retrieving objects from the floor	2.0	2.7	3.5	3.8	4.0	4.0
Turning trunk (feet fixed)	2.1	2.7	3.4	3.8	3.9	4.0
Standing with feet together	1.7	2.5	3.3	3.7	3.9	4.0
Reaching forward while standing	1.9	2.5	3.1	3.4	3.7	3.8
Turning 360°	1.0	1.6	2.4	3.0	3.5	3.8
Stool stepping	0.8	1.4	2.2	2.8	3.4	3.8
Tandem standing	1.0	1.5	2.0	2.5	3.1	3.5
Standing on one leg	0.4	0.6	0.9	1.5	2.4	3.1

The item reference profile is the expected score for each sub-item in the BBS of the subjects belonging to each rank. The values Rank 1–6 represent each rank in the model of a six-rank model of not-specified distribution.

BBS, Berg Balance Scale

Each rank and its number of FACs are presented in Table 4, which shows that the higher the rank, the more likely it is that someone with a higher FAC belongs to it. The correlation coefficient between rank and FAC was strong at rs = 0.81 (p <0.01).

**Table 4.** FACs for each rank

		Rank 1	Rank 2	Rank 3	Rank 4	Rank 5	Rank 6
FAC	0	17	0	0	0	0	0
	1	5	0	0	0	0	0
	2	10	3	2	0	0	0
	3	8	8	32	21	7	5
	4	1	0	10	32	23	41
	5	0	0	0	1	4	63

FAC, functional ambulation categories

## Discussion

We used LRT to analyze the number of ranks of balance ability in patients with stroke when the BBS is used. Most of the subjects were in the phase of early subacute stroke^19)^. The balance function of the patients could be graded in six ranks, and each rank had different characteristics of balance function. Our analyses also revealed that rank was strongly correlated with the degree of independence in walking related to balance function, indicating external validity. The results provide useful information to guide the characterization of the subject’s balance impairment and the therapy plan that should be a prioritized focus in improving balance function. Clinicians can determine the rank of a stroke patient’s balance ability to determine which BBS sub-items need improvement and by how many points to achieve one higher rank in balance ability. This is useful information for setting up exercises of the appropriate category and difficulty grade.

We observed that the test reference profile scores increased with rank, but the extent of the increase varied between ranks. Most of the patients in this study were older adults (mean age = 72.4), and the MDC of the BBS in older adults is 4 points if the patient’s initial BBS score is between 45 and 56; 5 points if it is between 35 and 44; 7 points if it is between 25 and 34; and 5 points if it is between 0 and 24^29)^. The difference in scores from ranks 1 to 4 that were revealed in the present analyses is thus considered to be a change beyond measurement error. The difference in scores between ranks 4 and 5 was 3.8 points, and the difference in scores between ranks 5 and 6 was 2.0 points. However, the BBS’s MDC for patients with stroke who can walk on level ground and for whom outpatient physiotherapy can be carried out is 2.7 points^30)^. Most of the patients with rank 4 and above are in FAC category 4 and above and can walk independently on level ground. The measurement errors obtained herein are thus close to those of patients whose balance function allows them to undergo physiotherapy at home as an outpatient, and we consider the influence of measurement errors to be minimal.

Our results demonstrated that it is possible to characterize the balance function of each rank from the test reference profile, item reference profile, and the degree of walking independence. The patients’ test reference profiles were calculated from the distribution of scores on the BBS sub-items and compared to previously reported cutoff values and the data in Table 4 for clinical meaningfulness. The cutoff values for falls and slow walking in stroke patients were 47.0 and 47.5, respectively^31^^,^^32)^. Therefore, many patients below rank 3 (test reference profile = 43.4) were unable to walk independently because they were below the cutoff values for the BBS associated with walking independence. On the other hand, patients with rank 4 (test reference profile = 48.1) and above were above the cutoff value and, therefore, able to walk independently on level ground. Ranks 5 and 6 have test reference profiles above 51 points, which is also above the cutoff for predicting falls in older people living in the community and community walking in patients with stroke^10^^,^^33)^. This suggests that post-discharge, many patients with stroke can engage in activities outside the home. However, there was some variation in the FAC values corresponding to each rank; in particular, FAC = 3 was present at all ranks. This is because not only BBS but also cognitive function and walking speed are influential in determining walking independence^34)^. In particular, because some subjects were using walking aids, it is thought that some subjects had low BBS scores but high cognitive function and increased their walking independence using appropriate aids and adjusting their walking speed. Conversely, people with high BBS scores but low cognitive function and walking speeds that were too slow or too fast would have needed to be supervised.

The present item reference profile results showed a characteristic balance impairment in each rank. A patient in rank 1 has a sitting-to-standing item reference profile of 2.7 points and can change posture to standing on his/her own but can only hold the standing posture for a short time. However, the item reference profiles for retrieving an object from the floor, turning one’s trunk (feet fixed), reaching forward while standing, and standing with feet together are >1 point, which means that items involving a shift in the center of gravity can be carried out under supervision. This suggests that an increasing number of individuals can engage in walking practice at short distances with assistance and supervision (Table 4). Rank 2 had an item reference profile of >3 points for sitting to standing, standing unsupported, and transfers, plus an item reference profile of >2 points for turning the trunk, reaching forward while standing, and standing with feet together. This means that patients at this rank have the necessary lower limb support and static standing balance to stand stably, as well as the ability to shift the center of gravity within the base of support and to correct the position of the feet by him/herself. Rank 2 is therefore considered to indicate being able to walk with less assistance or supervision (Table 4).

Rank 3 has an item reference profile of ≥3 points for static posture maintenance and items that involve shifting the center of gravity within the base of support but an item reference profile (IPR) of <3 points for turning 360°, tandem standing, and stool stepping. A patient at rank 3 thus has adequate ability to maintain a standing position and to shift the center of gravity within the basal plane of support; however, the patient’s ability to shift the center of gravity outside the basal plane of support requires supervision or is inadequate. It is thus likely that many of these patients will require supervision for ambulation (Table 4). Rank 4 has an IPR of ≥3.5 points, with the exceptions of reaching forward while standing, turning 360°, tandem standing, stool stepping, and standing on one leg. This means that the static standing position of a patient at this rank is stable, and the center of gravity can be shifted outside the base of support with greater speed. However, the item reference profiles of 2.8 points for stool stepping and 1.5 points for standing on one leg suggest that a patient at this rank is not able to walk independently on stairs or outdoors (Table 4).

Rank 5 has an item reference profile of 3.4 points for stool stepping and an item reference profile of 2.4 points for standing on one leg, which means that the patient can stand on one leg for a short time and lift his/her leg over a step. It is thus considered possible for the patient to raise hir or her feet over steps and walk outdoors (Table 4). Rank 6 has an item reference profile score of 3.1 points for standing on one leg and an IPR of ≥3.5 points for the rest of the BBS items. Many patients at this rank can thus be expected to be able to achieve independence for walking outdoors because of (i) their sufficient center of gravity shift outside of the supportive basal plane and (ii) supportive lower limbs that enable them to maintain their posture even on one leg (Table 4).

The results of this study demonstrate the test reference profile, which is the average score for each rank of balance ability, and the characteristics of balance function for each rank. Clinicians can estimate the rank of the balance function to which a patient belongs based on the test reference profile score closest to the patient’s BBS score. In addition, a program of intervention can be determined by comparing the item reference profiles of the BBS sub-items of the estimated ranks with the patient’s BBS sub-item scores. If there is an item for which a patient’s BBS sub-item score is not above the estimated rank item reference profiles, then that item is considered for the therapy. In cases where a patient’s BBS sub-item scores are all higher than the estimated rank item reference profile scores, a comparison is made with the item reference profile scores of one higher rank. The items for which the BBS sub-item scores are lower than the item reference profile scores are then considered for therapy. Clinicians can thus easily estimate the rank and determine the therapy strategy based on the BBS evaluation. Hence, the results of this study are useful for characterizing balance impairments in patients with stroke and in planning intervention strategies.

In addition, when therapy is aimed at improving walking independence, the time spent on balance function exercises could be made more appropriate by comparing rank with walking independence. People with a low balance function rank but high walking independence (e.g., rank = 1, FAC = 3) may further improve their walking independence if they focus on balance function practice. However, people with a high rank but low walking independence (e.g., rank = 6, FAC = 3) may find it difficult to improve their walking independence even if they focus on balance function exercises.

The strengths of this study are (i) the identification of the type of balance impairment that generally occurs in individuals who have experienced a subacute stroke and (ii) the identification of the process of improving balance function from non-independent walking to gait function that enables community living. These results will clarify what clinicians need to focus on to improve their patients’ balance function. In addition, the differences between the item reference profiles of each rank obtained in this study and the BBS sub-item-scoring trends of patients to be treated can be understood as a functional impairment characteristic of the patient. This can be used as a guideline for deciding on a treatment strategy.

### Limitations

This study has several limitations. Its design was retrospective, and the patient population criteria did not exclude patients with cognitive or higher brain dysfunction, which may affect balance function. Although the external validity of the BBS balance ranks calculated by LRT was verified by the FAC test, balance and walking independence contain different constructs. Further studies are thus necessary to clarify the differences in the various components of balance, such as differences in the sway of the center of gravity for different balance ranks. In addition, because we did not have information on the patient’s walking ability before their stroke, our population may have included individuals whose walking function was impaired before the stroke. Although using LRT allows the calculation of the rank membership profile, which indicates the probability of belonging to each rank for each individual, we have not yet been able to implement interventions using data from individuals outside the dataset used in the present analyses.

## Conclusion

We used LRT to identify the balance impairment ranks of the BBS in patients with subacute stroke, to identify the characteristics of balance impairment in each rank, and to examine the relationship between balance impairment and walking ability. The results demonstrated that when the BBS was classified into six ranks, there were different functional impairments in each rank, and each rank was related to walking ability. The findings of this study help clinicians understand balance impairments in patients with stroke and aid in planning therapeutic strategies.

## Funding

This work was supported by the Japan Society for the Promotion of Science (JSPS) KAKENHI (grant no. JP21K17458).

## Conflicts of Interest

The authors declare no conflicts of interest.
